# Transcriptional insights into pathogenesis of cutaneous systemic sclerosis using pathway driven meta-analysis assisted by machine learning methods

**DOI:** 10.1371/journal.pone.0242863

**Published:** 2020-11-30

**Authors:** Xiao Xu, Meera Ramanujam, Sudha Visvanathan, Shervin Assassi, Zheng Liu, Li Li

**Affiliations:** 1 Computational Biology, Boehringer-Ingelheim Pharmaceuticals Inc, Ridgefield, CT, United States of America; 2 Immunology and Respiratory Diseases Research, Boehringer-Ingelheim Pharmaceuticals Inc, Ridgefield, CT, United States of America; 3 Translational Medicine and Clinical Pharmacology, Boehringer-Ingelheim Pharmaceuticals Inc, Ridgefield, CT, United States of America; 4 Division of Rheumatology, University of Texas Health Science Center at Houston, Houston, TX, United States of America; Indiana University Purdue University at Indianapolis, UNITED STATES

## Abstract

Pathophysiology of systemic sclerosis (SSc, Scleroderma), an autoimmune rheumatic disease, comprises of mechanisms that drive vasculopathy, inflammation and fibrosis. Understanding of the disease and associated clinical heterogeneity has advanced considerably in the past decade, highlighting the necessity of more specific targeted therapy. While many of the recent trials in SSc failed to meet the primary end points that predominantly relied on changes in modified Rodnan skin scores (MRSS), sub-group analysis, especially those focused on the basal skin transcriptomic data have provided insights into patient subsets that respond to therapies. These findings suggest that deeper understanding of the molecular changes in pathways is very important to define disease drivers in various patient subgroups. In view of these challenges, we performed meta-analysis on 9 public available SSc microarray studies using a novel pathway pivoted approach combining consensus clustering and machine learning assisted feature selection. Selected pathway modules were further explored through cluster specific topological network analysis in search of novel therapeutic concepts. In addition, we went beyond previously described SSc class divisions of 3 clusters (e.g. inflammation, fibro-proliferative, normal-like) and expanded into a much finer stratification in order to profile SSc patients more accurately. Our analysis unveiled an important 80 pathway signatures that differentiated SSc patients into 8 unique subtypes. The 5 pathway modules derived from such signature successfully defined the 8 SSc subsets and were validated by *in-silico* cellular deconvolution analysis. Myeloid cells and fibroblasts involvement in different clusters were confirmed and linked to corresponding pathway activities. Collectively, our findings revealed more complex disease subtypes in SSc; Key gene mediators such as IL6, FGFR1, TLR7, PLCG2, IRK2 identified by network analysis underscored the scientific rationale for exploring additional targets in treatment of SSc.

## Introduction

Systemic sclerosis (SSc) is a heterogeneous autoimmune disease characterized by the dysregulated immune system, vasculopathy, and systemic fibrosis of the connective tissues. SSc is a rare disease with a prevalence around 240 patients per million in USA [1]. Still, 30% of patients died within 10 years of diagnosis. Many pathological factors were found to contribute to the disease progression, including dysregulated innate and adaptive immune system, inflammatory mediators, fibroblasts dysfunction, and other components such as over-disposition of extracellular matrix and the microvascular endothelial cells (MVEC), as well as fibro-proliferative vasculopathy of small vessels [2, 3]. SSc disease, characterized by skin thickening on body surface, may progressively lead to severe damage of multiple internal organs such as digestive system, heart, lung or kidneys. Based on the extent of skin involvement, the type of autoantibody, and the pattern of organ involvement, SSc is commonly described as two clinical categories: diffuse SSc (dSSc) and limited SSc (lSSc) [4–6].

It is well accepted that both genetic and environmental factors contribute to the pathophysiology of SSc. Genome-wide association studies (GWAS) have identified some key genetic associations that confer susceptibility to SSc including major histocompatibility complex (MHC) II region as being the most significant in this disease [7–9]. Toll-like-receptors (TLR), such as TLR 2, 3, 4, 7, 8, and 9, IRF5, STAT4, HSP70, HMGB-1, and CD169 are believed to have particular relevance to SSc pathogenesis as well [10–12]. TGF-β1, a pluripotent cytokine produced by most cell types, and IL-4, a characteristic cytokine from Th2 cells and mast cells were primarily studied for their roles in stimulating collagen synthesis and chemotaxis by fibroblasts during SSc onset [13, 14]. Meanwhile, a plethora of other cytokines, chemokines and growth factors, such as IL-5, IL-6, IL-13, IL-18, IL-33, MCP-1, PDGF, and CTGF, have been found to associate with SSc pathogenesis by previous studies [15, 16].

The disease of SSc is heterogeneous both clinically and genetically due to the involvement of multiple pathogenic pathways. Thus, a better understanding of the interplay of these factors is considered necessary in developing novel therapies for improved treatment options. Extensive efforts have been made to define and comprehend the disease mechanism at molecular level. Several microarray analysis studies or recent meta-analysis have delineated the existence of a few major key subtypes of SSc skin samples such as immune response dominant, fibro-proliferative and normal phenotypes [17–21]. This approach was applied in recent trials to define the responder populations based on the basal gene expression pre-treatment. Some of the potential novel therapeutic concepts were also proposed from these studies to target specific biological pathways [18]. However, most of the studies either analyzed the gene expression datasets with limited patient sample size,or relied on multi-source meta-analysis with suboptimal integration strategy. Challenges of the latter became more evident when the total number of public SSc studies is limited given its rarity, and involved a wide variety of microarray platforms using diverse probe designs. Researchers adopted meta-analysis when individual study is underpowered; meanwhile, they need to come up with a strategy to integrate data sets of varying sources. Typical solutions include either incorporating data by gene weights (e.g. p values) of each study, or merging data all together through strong normalization procedure. Both methods may possess drawbacks when the scope of commonly shared genes is limited, or strong data source effects exist. To address these potential issues, our study adopted a different approach combining gene expression data: we surveyed public genomic data sets with a novel application of gene-pathway space projection using GSVA algorithm [22]—A non-parametric, unsupervised gene set enrichment method that calculates sample-wise pathway enrichment scores based on gene expression within the sample. This procedure was followed by consensus clustering and machine learning assisted feature optimization process to explore patients’ heterogeneity. Our analysis identified disease subtypes on pathway space directly, resulting in a diversified classification of SSc samples into more pertinent subgroups. Furthermore, cluster specific Protein-Protein-Interaction (PPI) network analysis and cellular deconvolution procedure were used to reveal important gene regulators as emerging therapeutic target in SSc treatment.

## Methods

### SSc study search and data collection

All of the 9 SSc data sets were obtained by searching on 2 popular gene expression repositories: GEO or ArrayExpress databases, using key words “systemic sclerosis”, “systemic scleroderma” or “SSc”. We looked into studies published in the last 12 years and focused exclusively on microarray data. Next generation sequencing or other types of high throughput gene expression data were not considered since the absolute majority of public SSc datasets were based on microarray, and it is computationally more consistent to combine data sets of the same platform for meta-analysis. During the initial search, a total 26 SSc studies were identified from the two data repositories. They were subsequently screened for redundancy to exclude 3 studies sharing the same set of samples with others. An additional filter was applied to remove another 12 studies of non-skin samples. Lastly, we disqualified two studies (GSE9285 and GSE76806) as these data sets share much less common genes with others. The final list of studies included in our current investigation is specified in **S1 Table**. Many of the studies we selected were clinical trials that involve medical intervention on patients; other studies have paired biopsy position (e.g. forearm vs back) design wherein non-involved skin area was sampled. We generally only considered baseline SSc patients and focused exclusively on involved (affected) skin samples on forearms to avoid any confounding factor. The availability of clinical information such as disease stage, severity and internal organ involvement varied a lot across studies, we hence concentrated on the factor of major disease phenotypes (dSSc, lSSc & Controls) in our research. Overall, we had a total of 141 SSc samples and 80 controls entered our meta-analysis, including 126 dSSc and 15 lSSc; the detailed sample distributions are listed in **S2 and S3 Tables**.

### Pathway compendium formation and filtering

From each GEO study, the normalized gene matrix data (uploaded by their original authors) was carefully examined using PCA and boxplot profiling. We then applied GSVA algorithm [22] (R package GSVA v1.34.0) to transform gene expressions to pathways scores by querying against Metabase^TM^ [23, 24], a commercial database from Clarivate Analytics. The pathway enrichment matrices of each individual study were then merged into one compendium (937 pathways × 221 samples). PCA based QC procedure was performed on this joint pathway matrix to check batch effects and detect outliers. Two filter steps were subsequently applied: 1) we selected several key words (“JAK-STAT”, “PI3K”, “TGF-beta”, “WNT”, “extracellular matrix”, “VEGF”, “Hedgehog” and “Notch”) from previously reported SSc associated pathways [18, 25–27] to search against Median Absolute Deviation (MAD) score sorted (decreasing) Metabase^TM^ entries using an incremental size of 50. The criterion was to include at least one pathway that contained a given key word when we searched from top to bottom. 2) We only kept the pathway that has enrichment scores over 3 in at least 5 samples out of 141 cases. In the end, a total of 693 pathways passed the filter and entered subsequent analysis.

### Consensus clustering and clusters number determination

Consensus clustering procedure was performed using R package ConsensusClusterPlus (V 1.50.0) based on 90 percent of the samples, Euclidean distance and ward.D metrics. Optimal number of clusters were determined by visual inspection of consensus matrix pattern, CDF plot and delta plot from default function output as well as the plot of an external metrics: silhouette [28] (R package Cluster V2.1.0). Per the focus of current study, we primarily concentrated on cluster numbers greater than 4 in the process. Collectively, we considered all metrics supporting N = 8 as the final optimal number of clusters in our SSc stratification.

### Choosing machine learning classifier

In order to find the best classifier for pathway feature selections, we surveyed a group of multiclass machine learning classifiers from R package caret (v 6.0–84) [29]. These methods were: Single C5.0 Tree (C5.0Tree), Gradient Boosting Machines (gbm), K-nearest neighbors (kknn), Naïve Bayes (nb), Partial least Squares (pls), Random Forest (ranger), Shrinkage Discriminant Analysis (sda), Stabilized Linear Discriminant Analysis (slda), Support Vector Machines Radial kernel (svmRadial) and Bagged CART (treebag). We first attempted reconstructing the 8 SSc clusters with each algorithm using all 693 pathways and a 10 fold cross-validation procedure. Subsequently, top 50 and top 100 pathways were selected using recursive feature elimination method (function rfe from caret) and repeated the classification step. For each classifier, 5 random levels of main parameters were tried (tuneLength = 5 in R Caret function) in training process. The highest accuracy of each run was recorded and compared accordingly.

### Random forest based feature selection and classification

Fast implementation of random forest method (ranger) [30] was chosen for pathway feature selections. We first ran ranger using all 693 pathways with repeated (n = 5) 10 fold cross-validation and reached an overall accuracy of 83.6% (tunelength = 5). The pathway features were ranked by gini index at the same time. A looping process was then setup to survey from top 10 to top 150 pathways (incremental by 10) using the same ranger classification and a tunelength = 5. When top 80 pathways were used the overall classification accuracy (82.8%) was roughly at the same level as that achieved by all pathways. Heatmap was generated (R pheatmap V 1.0.12) to display the 80 pathway signature across 141 samples plus control and Euclidean distance based clustering was also performed at pathway level. By visual inspection, a cutoff resulting in 5 pathway modules (Blue, Red, Green, Yellow and Black) was defined and used for subsequent cluster functions interpretations (**Table 1**). In each cluster & pathway-module combination, the average of pathway enrichment scores was calculated (shown in **S4 Table**) and the value range was divided into 3 levels: High (> = 0.15), Moderate (-0.15~0.15), and Low (= <0.15) to reflect the pathway enrichment levels in each cluster and pathway module.

**Table 1:** Pathway module function and cluster enrichment pattern.

Pathway Modules ^a^	Black	Yellow	Blue	Red	Green
**Pathway Name**	Metabolism-1	Metabolism-2	Immune-Fibrosis	Immune Response-2	Immune Response-1
**# of pathways**	14	7	23	18	18
**# of genes**	151	249	650	475	567
**Clusters**	**Enrichment Level** ^**b**^				
**Cluster 1**	High	High	Low	Moderate	Low
**Cluster 2**	Low	Moderate	Moderate	High	High
**Cluster 3**	Low	Moderate	High	Low	Moderate
**Cluster 4**	Low	Low	High	High	High
**Cluster 5**	High	High	High	High	High
**Cluster 6**	High	High	Low	Low	Low
**Cluster 7**	Low	High	Low	Moderate	Low
**Cluster 8**	Moderate	Low	High	High	High
**Control**	Moderate	Moderate	Low	Low	Low

^**a**^ The pathway modules are defined by the functions of majority of the pathways in the module as well as the module specific cellular components (See **Results**: Cell type signature profiling part in SSc clusters).

^b^ A three level system (High, Moderate, Low) was adopted to denote the pathway enrichment level of the 8 SSc clusters and control cohort based on average enrichment scores (See **Methods** for details).

### Cluster specific network analysis

We extracted genes corresponding to the 5 pathway modules respectively (**Table 1**) and performed two nodes prioritization algorithms (random walk and neighborhood scoring [31, 32]) on each of the pathway set using those genes as start nodes. The algorithms were implemented through CBDD package [33] and queried against Metabase^TM^ network. Random Walk restart probability was set at 0.75 and neighborhoodScoring alpha was set as 0.5. The final targets from both programs were ranked by their hub scores (separately) for each pathway set. In case of target list having more than 500 genes, only top 500 genes were used to cross-reference with MRSS in the following step.

### MRSS and gene correlation

Modified Rodnan skin score (MRSS) was extracted from one study from GEO. We then calculated Pearson correlation coefficients for each gene with MRSS based on quantile normalized gene expressions. MRSS associated genes were selected based on p value < = 0.05 and correlation coefficient > 0.

### Cellular deconvolution in pathway modules

We selected common genes across all 9 SSc studies and combined individual gene expression matrices into a gene level compendium matrix (12306 genes by 141 samples). Combat method [34] (R package sva v3.20.0) and quantile normalization (R package preprocessCore v1.34.0) were applied to remove batch effect and baseline differences across samples. We then used GSVA algorithm to calculate cell type enrichment using gene markers curated by a previous study [35] for fibroblasts, endothelial cells and macrophage, and an internal 9 genes list (CD14, MS4A6A, IL1b, CSF1R, C1QA, C1QB, MS4A7, HCK, C5AR1) for myeloid calculations. Pairwise Welch’s T test was also performed to compare the cell type enrichment level between each pair of the clusters.

## Results

**Fig 1:**
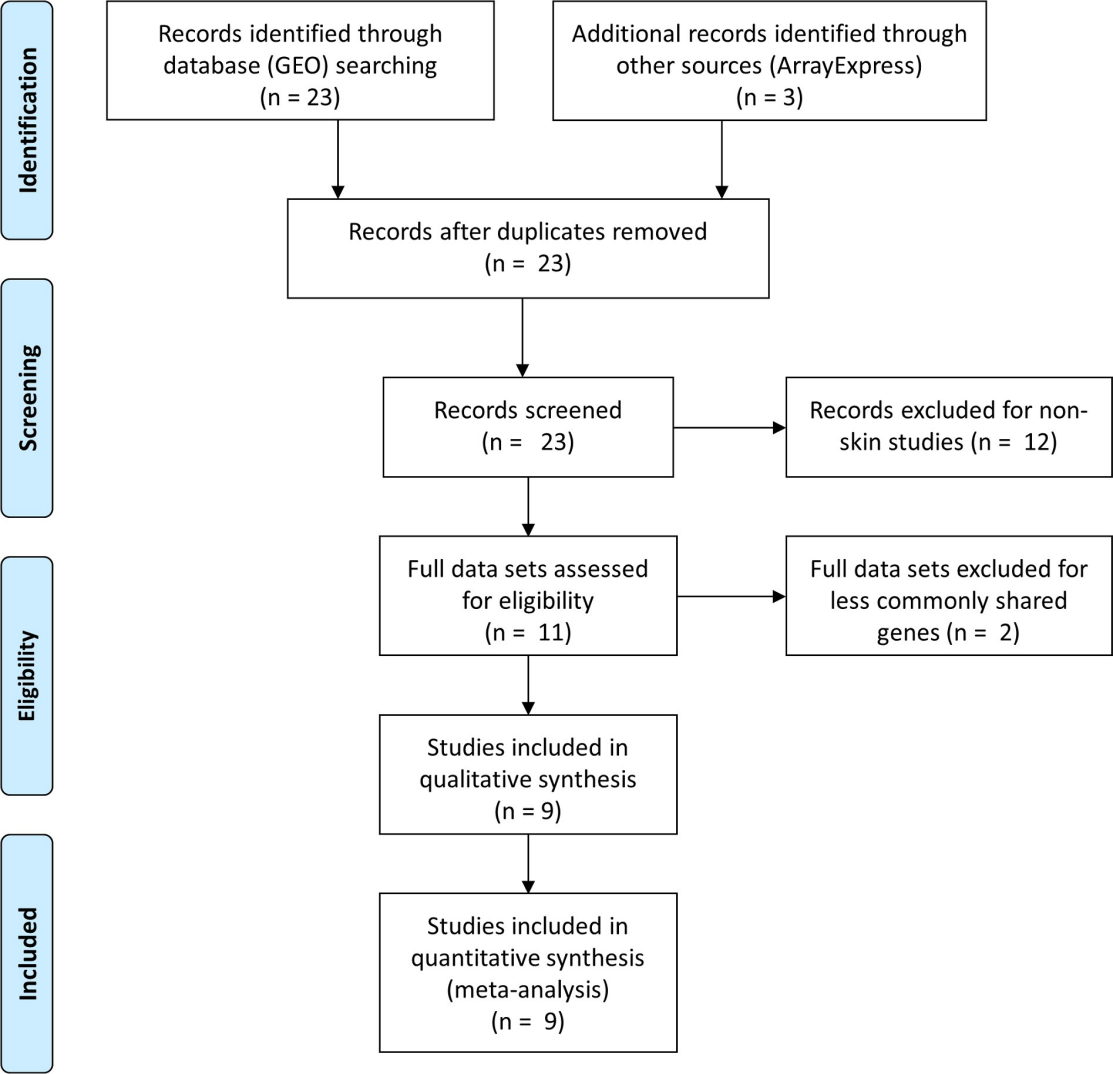
Flow diagram of studies collection and screening process. An initial selection of 26 SSc studies from both GEO and ArrayExpress data repositories were screened for sample redundancy (to exclude studies sharing the same set of samples), biopsy sites (studies of non-skin samples excluded) and commonly shared genes (2 data sets removed) to arrive at the final 9 SSc studies for the final meta-analysis.

We surveyed PubMed and related GEO database of the past 15 years (2004 ~ 2019), selecting 9 relevant skin SSc studies through a multi-step screening and filtering process (**Fig 1**) for the final meta-analysis. With a novel application of gene-pathway space projection, we sought to unify different SSc studies into a single pathway-based compendium before further dissecting their molecular signatures. Consensus clustering procedure was then applied to classify SSc samples into more discrete subgroups than previously documented 3 or 4 clusters [18, 20, 21]. Overall, our approach leveraged the SSc pathway activation pattern and extended into underlying gene regulator discovery through network analysis.

**Fig 2:**
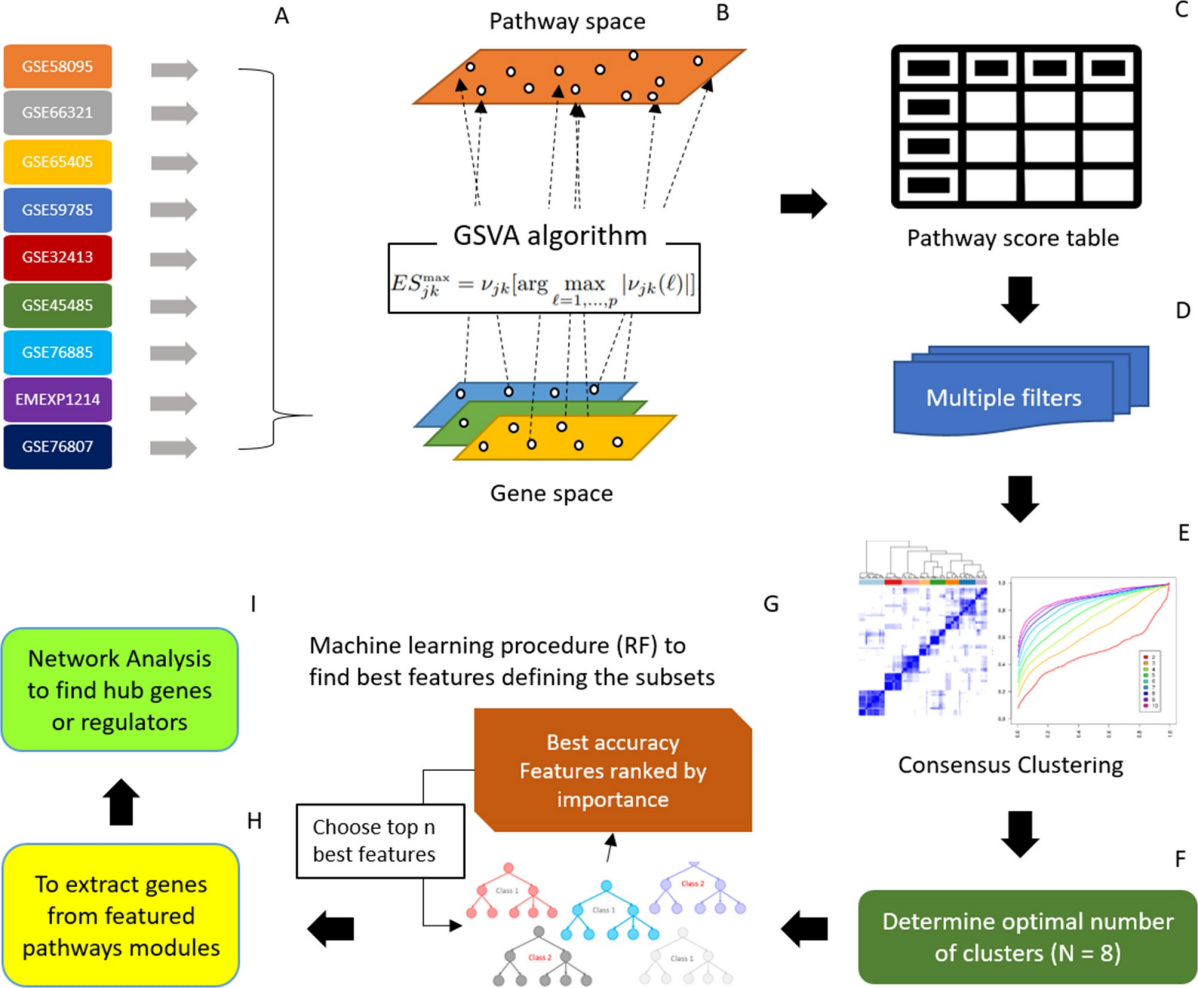
Schematic view of data processing and analysis. (A) 9 SSc studies were used in the current meta-analysis. (B) Preprocessed gene expression matrix of each study was projected into pathway space using GSVA algorithm (C) 9 SSc studies were projected into one pathway enrichment table (D) Multiple filters were applied to remove pathways of constant enrichment scores. (E) Consensus clustering procedure was applied and (F) optimal number of subsets were determined. (G) Machine learning procedure was used to determine best pathway modules at maximum accuracy. (H) Genes were extracted from selected top pathways modules that differentiate SSc subtypes and (I) subsequent network analysis was applied to select important gene regulators behind each subset.

The first major challenge in gene expression meta-analysis is to integrate information from each individual study of varying baselines and batch effects. We managed to address the issue by transforming individual gene expression into common pathway space using GSVA algorithm [22]. A consensus clustering procedure was then performed on the pathway enrichment compendium to identify 8 detailed SSc subtypes. To further refine the pathway features, we employed a random forest classifier to elect an 80 pathways set that achieved the same discriminant power using all pathways. In this manner, we were able to define the 8 SSc subsets based on the pathway signature, thereby enabling cluster specific gene hub explorations. Subsequent steps involved network analysis of each SSc subgroup and identification of latent gene regulators. The entire analytic pipeline, fully illustrated in **Fig 2**, featured a pathway-centered view of SSc patients’ heterogeneity and an objective data driven approach delineating the genomic differences that defined SSc pathogenesis.

### Unifying SSc studies by gene-to-pathway projection

Gene expression from individual study was transformed into pathway enrichment scores, and subsequently merged into one joint pathway compendium. We performed PCA on the compendium and found no potential batch effect or any outlier sample (**S1 Fig**). Similarly, we failed to observe notable difference among diffuse, limited SSc samples and Controls, suggesting that the clinically groups may only differ on a subset of variable pathways. In addition, some of the disease samples were known to be highly similar to controls as documented by previous studies [19, 21].

Moderate filter steps based on Mean Absolute Deviation (MAD) and enrichment scores were applied to remove uninformative pathways. Relatively lenient cutoffs (See **Methods** for details) were used in this process so that we did not exclude any interesting biological feature. As a result, a sum of 693 pathways passed the filters and entered downstream clustering analysis.

### Detailed stratification of systemic sclerosis patients based on pathway enrichment

**Fig 3:**
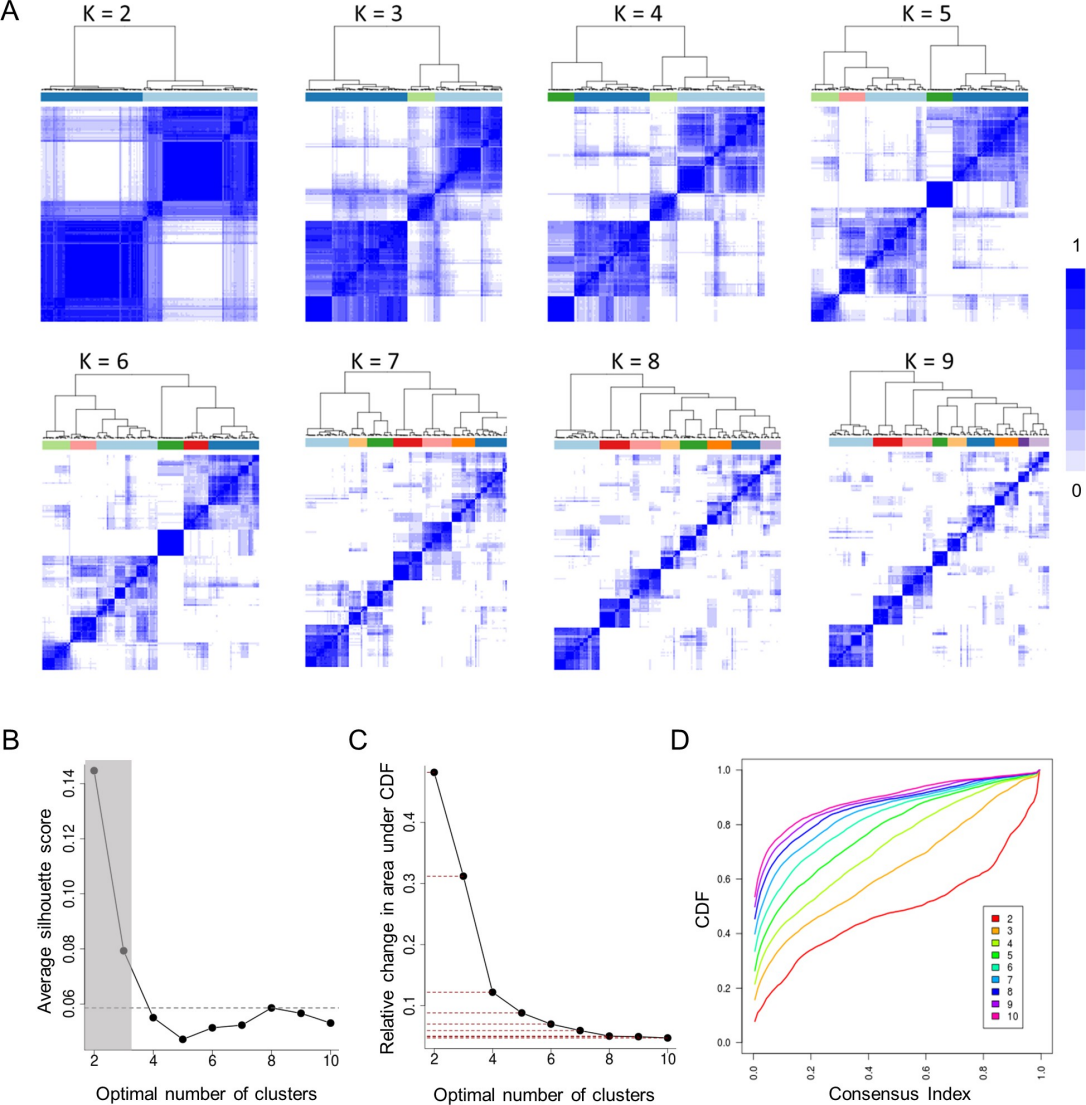
The determination of optimal number (K) of detailed SSC subsets. (A) Heatmaps of consensus matrices for K (number of clusters) ranging from 2 to 9 showing clear-cut consensus clustering pattern at K = 8. (B) Silhouette plot showing average silhouette score peaking at K = 8 when considering more than 3 clusters. (C) Delta area plot showing relative change in the area under the CDF curve with dotted line as reference. It is evident that from K = 8 the change of AUC of CDF becomes very minimal.(D) Consensus cumulative distribution function plot showing at cluster K = 8, the area under CDF curve almost maximized with little improvement when K increased to 9 or 10.

We performed consensus clustering [36] on 221 SSc samples across 11 studies based on previously filtered 693 pathways. To determine optimal number of SSc subsets, several consensus clustering metrics were used, including consensus matrices heatmaps, CDF function plot and Delta area plot (**Fig 3A, 3C and 3D**). Additionally, an external metric of silhouette scores was also generated (**Fig 3B**) to demonstrate the optimal cutoff of SSc subtype numbers. Previous SSc studies mostly focused on 3 to 4 major SSc divisions [18, 20, 21, 37], featuring one or two sets as inflammation signature, a fibro-proliferative type and a normal-like group. Our view of SSc heterogeneity, nonetheless, differed from such granular classification and pushed into a finer division of more than 4 classes. We therefore focused on pattern fuzziness (e.g. consensus matrices heatmap), AUC (CDF plot) or line elbow point (e.g. silhouette plot) where number of clusters is greater than 4 (**Fig 3A–3D**). Eventually, we selected the optimal cluster number at N = 8 to strike a balance between minimal cluster size and a good stratification setting of SSc patients. Details on the cutoff determination methods of the 4 plots are specified in Methods section. The 8 subtype classification of 141 SSc patients defined a spectrum of disease clusters ranging from the biggest cohort of 28 (19.8%) samples to the smallest cohort of 8 (5.67%) patients (**S2 Table**). It was also noted that none of the clusters is significantly enriched with an individual SSc study; the overall association between cluster membership and GEO study sources is non-significant (Chi-square test: p = 0.15). In addition, neither dSSc nor lSSc patients was found to enrich a certain subtype (Chi-square test: p = 0.12), although some level of cluster associated lSSc depletion was observed in cluster 2 and cluster 4 (**S3 Table**). Overall, the 8 SSc clusters were established independent of study effects or clinical phenotypes (dSSc vs lSSc).

### Delineation of a predictive pathway subset for SSc patients’ classification

**Fig 4:**
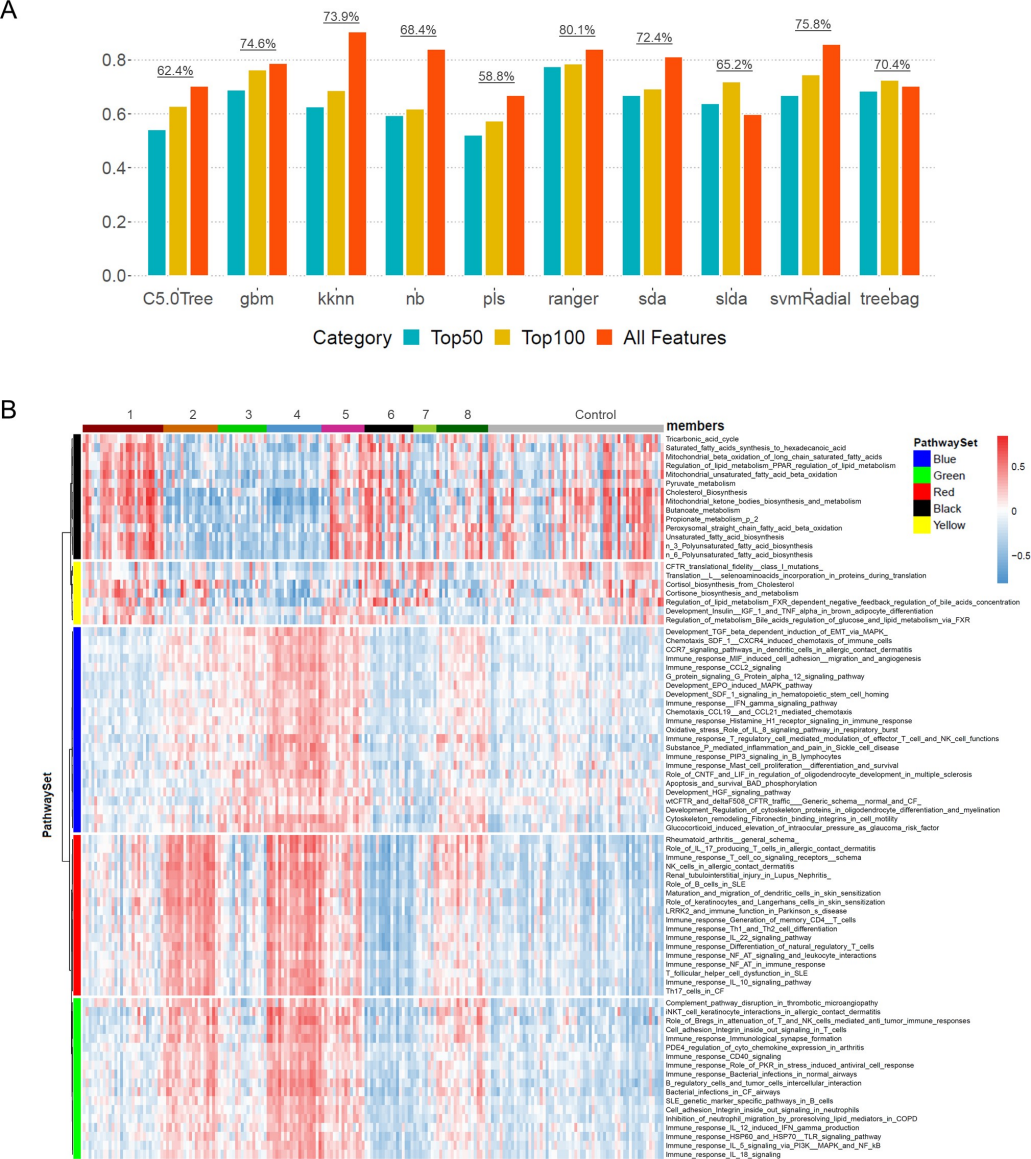
The choice of best supervised machine learning algorithms to classify SSc pathway set into 8 clusters / subsets. (A) Different supervised classification methods were evaluated using all features, top 50 features and top 100 features by recursive feature elimination process. (B) The heatmap of 8 SSc subsets was generated based on top 80 pathways selected by random forest method according to Gini index. The 8 subsets are later defined respectively by the pathways they are enriched in. Subsequently 5 pathway modules were defined by tree-cut with visual assistance so that their average levels of expression can determine the distinct features of the 8 clusters.

A total of 693 pathways, many of which redundant, were used to establish the 8 cluster SSc classification. The sheer number of pathways involved in consensus clustering actually hampered our effort in deriving meaningful biological interpretations, not to mention questing relevant gene targets. In addition, we arrived at the 8 SSC subsets through a consensus clustering approach based on Euclidean distance metrics and the robustness of their cluster memberships may need further validation by a different method. To address these issues, we adopted a machine learning procedure aiming to reconstruct the cluster membership and reduce the number of pathways through an accompanying feature selection process. The goal of such process was to find a highly predictive pathway subset that can achieve the same level of classification accuracy as that of all 693 pathways. We evaluated a diverse set of popular multiclass machine learning algorithms based on 10-fold cross-validation of the training set (See **Methods**). A couple of algorithms reached more than 80% accuracy using all 693 pathways, however, only gbm, ranger and svmRadial could roughly uphold accuracy level when we rerun the cross-validation using top 50 or 100 features (**Fig 4A**). Eventually, random forest (ranger) classifier was adopted since it on average achieved the highest level of accuracy in all 3 pathway modules (80.1%); In addition, it relied on a model based feature selection metric (Gini index) instead of random heuristic searching. We then adopted a step-up loop process using repeated cross-validation to determine an optimal number of top pathways that can reconstruct 8 SSc groups with comparative accuracy. As a result, a signature of top 80 pathways was elected whereby each SSc cluster can be interpreted biologically through its pathway enrichment pattern (**Fig 4B**). We also compared the profiles of all SSc subtypes with that of control; highlighting 5 pathway modules that can literally differentiate the 8 clusters by their expression level combinations (**Fig 4B**). It was readily evident that cluster 1 and 6 resembled control cohort, showing elevated level of cell metabolism pathways (Pathway-module Black). Meanwhile a minor difference was noted between the two subtypes that cluster 1 has slightly elevated level of immune response than cluster 6, suggesting a possible transitional role among cluster 1 samples. Such variation was also noted between cluster 2 and 4 wherein the average enrichment of Blue moderately differed between the 2 groups while both of them demonstrated a strong inflammation signature. Cluster 3, showing elevated level in Blue module only, formed a distinct cohort. Clusters 5 and 8 are again diverging solely on Pathway module Yellow although they are not so dissimilar to each other; both subsets demonstrated strong inflammatory, pro-fibrotic and cell-metabolism signatures. Lastly, cluster 7 appeared to be a separate “normal-like” cohort, enriching merely in a couple of metabolism pathways of Pathway module Yellow. Collectively, the 8 SSc cluster subtyping system was a more pertinent and finer stratification compared to the paradigmatic 3-type classifications (inflammatory, normal-like and fibroproliferative) by previous literatures [20, 21, 37, 38]. All pathway modules and their corresponding pathways are listed in **S5 Table**.

### Cluster specific network analysis unveils underlying gene regulators

After identifying 8 SSc subtypes using the pathway signature, we further explored the underlying gene interactions of each cluster through network analysis. The aim of such inquiry was to uncover latent gene hubs or upstream regulators that lay behind the pathway pattern; these genes, if established, could serve as potential drug target candidates for related SSc patient groups. To achieve this goal, we extracted all genes from each of the 5 pathway modules identified previously (**Fig 4B**) and used them as start nodes to query against the Metabase^TM^ biological network [24]. Two algorithms were used in the analysis: 1) random walk [32] and 2) neighborhood scoring [31]. Both methods traverse the undirected reference network from priori nodes, searching for hub genes that are significantly connected with start nodes while controlling for vertex promiscuity. Regulator or gene hubs identified in this process may or may not be part of start nodes, thereby expanded our scope into latent gene regulators.

**Fig 5:**
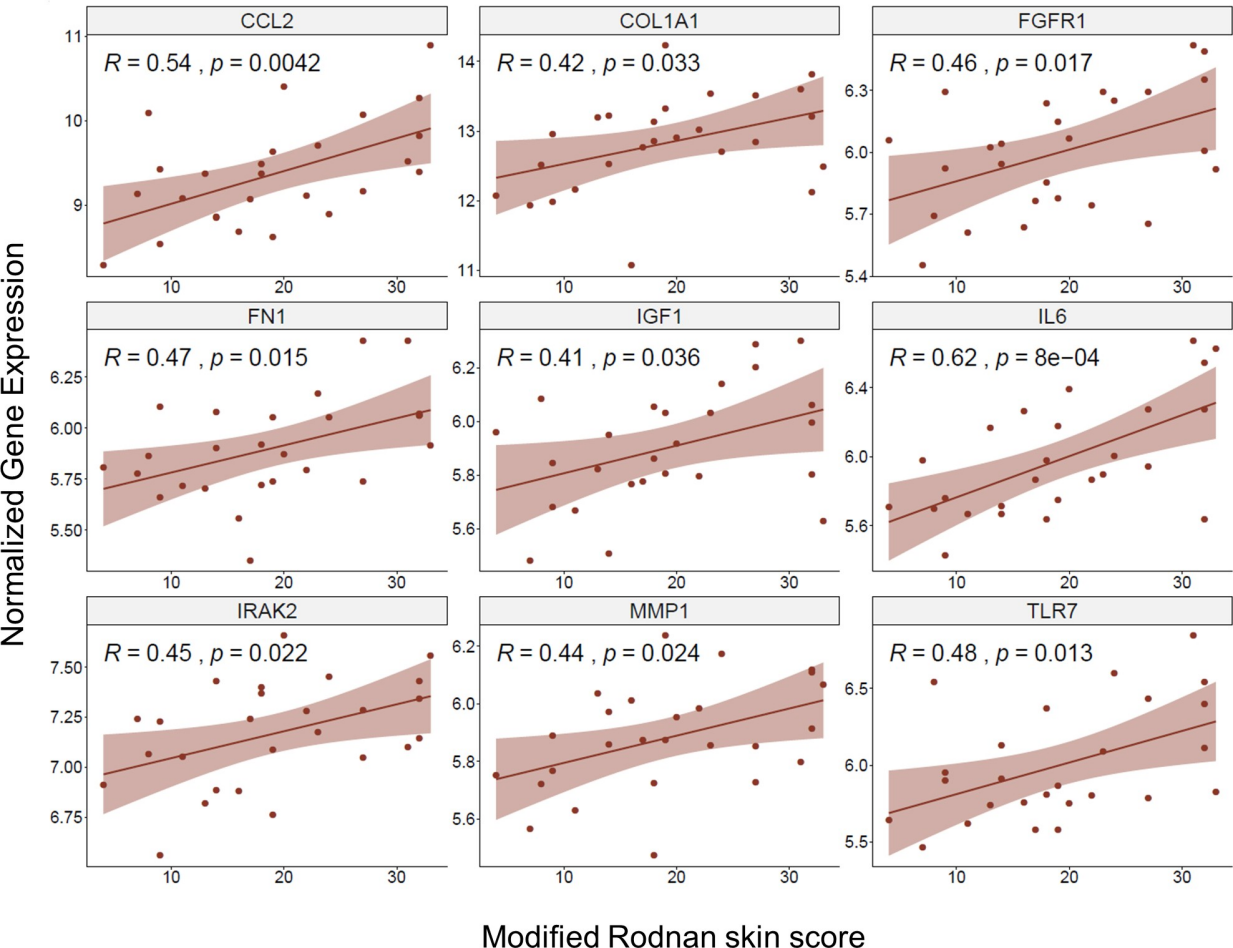
The Pearson correlation scatter plot of selected gene regulators showing significantly positive association between normalized gene expression and MRSS. The 9 genes (CCL2, COL1A1, FGFR1, FN1, IGF1, IL6, IRAK2, MMP1, TLR7) shown in the figure were selected from random walk or neighborhood scoring algorithm, and are positively correlated with MRSS based on study GSE58095. These 9 genes are mostly expressed on myeloid / stromal cells and many of them are deeply involved in cell skeleton remodeling, innate immune response and cell-cell adhesion processes.

Still, each algorithm led to hundreds of potential target genes ranked by their hub scores, we further reduced the target lists by overlapping with external clinical features such as modified Rodnan Skin Score (MRSS) which is an important clinical marker of SSc skin phenotype. We extracted MRSS from one study and correlated with genomic expressions. Genes significantly associated with MRSS were selected, and overlapped with the potential targets identified in pathway module based network analysis. This procedure generated a concentrated target list of 164 genes (**S6 Table**) based on 5 pathway modules, of which 92 unique genes emerge if we combine module sources. We picked a list of 9 genes (CCL2, COL1A1, FGFR1, FN1, IGF1, IL6, IRAK2, MMP1 and TLR7) based on their MRSS associations and biological assessment, showing their profiles in **Fig 5**.

**Fig 6:**
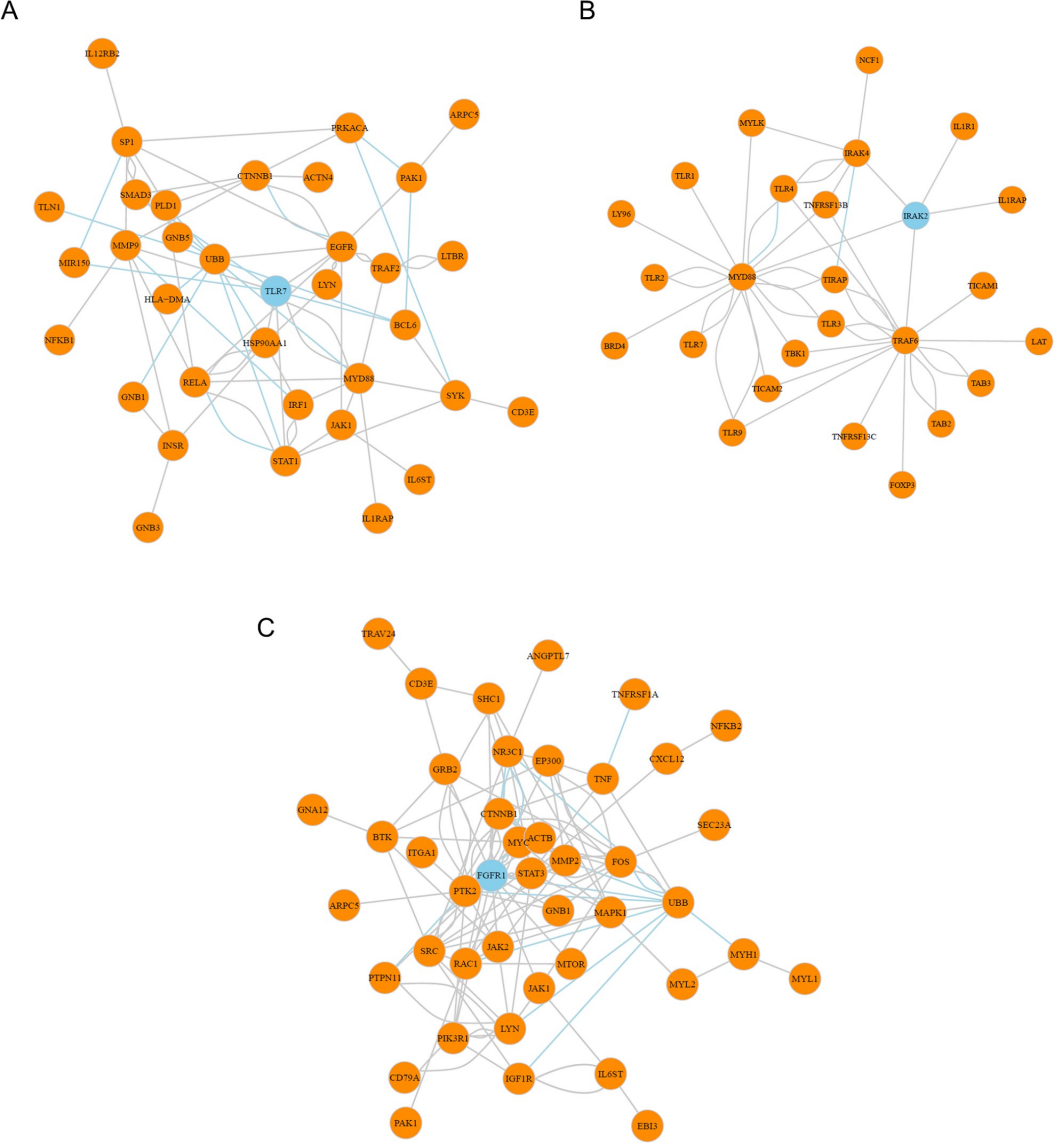
Hub-gene centered subnetwork based on random walk and neighborhood scoring algorithms. (A) TLR7 centered subnetwork identified by random walk algorithm and MRSS correlations. (B) IRAK2 centered subnetwork identified by neighborhood scoring algorithm and MRSS correlations. (C) FGFR1 centered subnetwork identified by random walk algorithm and MRSS correlations. In all 3 subnetworks, hub gene regulators are highlighted in blue and the edge of inhibiting relationships between two genes are colored in light blue as well.

We further focused on 3 genes of interest (FGFR1, TLR7, IRAK2) from the 9 gene list and investigated their subnetwork connections (**Fig 6**). Identified as key hub in its subnetwork, pursuing those three targets with small molecule compound or monoclonal antibody will possibly destabilize the topology of subnetwork, and normalize the aberrant biological pathways. A recent approval of Nintedanib (OFEV^®^) targeting FGFR for SSC-associated interstitial lung disease (ILD) substantiates our effort for drug targets based on PPI network analysis. Another possible target is IRAK2, a key serine/threonine kinase involved with IL1R and its downstream upregulation of NF-kB and its pathway. The function of IRAK2 in myofibroblast had been illustrated in a murine IRAK2 knockout model in which fibroblasts showed reduced reaction to multiple TLR ligands. In summary, our analyses has not only demonstrated some important biological pathways relevant to the disease, but also identified some important key hub genes/proteins within the biological pathway networks.

### Cell type signature profiling in SSc clusters

**Fig 7:**
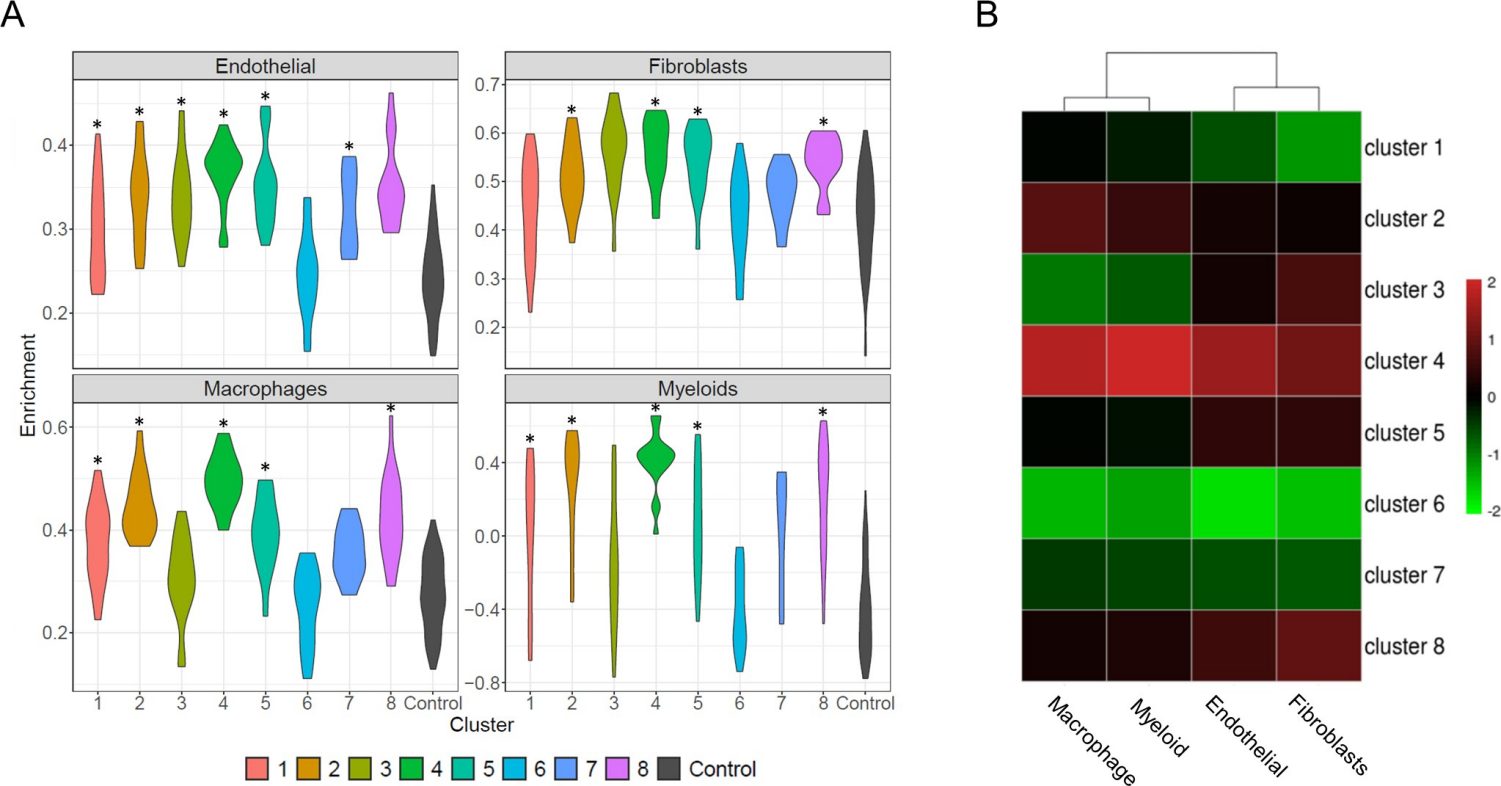
Cell type enrichment of 8 SSc clusters and comparison to control. (A) Violin plot showing 4 cell types (Endothelial, Fibroblasts, Macrophages and general Myeloid) enrichment among 8 SSc clusters as well as control cohort. Clusters significantly different (p value < = 0.05) from control group were marked with asterisk. (B) Heatmap showing relative enrichment score in the form of -log p value of t-test comparing 4 cell types against control.

When examining genes identified in network analysis, we recognized that many of them were highly related to myeloid cell and fibroblast signatures. A cell type enrichment analysis was then performed to profile fibroblasts and myeloid components in each cluster (**Fig 7A & 7B**). Endothelial cells and macrophages were also included in the analysis as a comparison. A follow-up pairwise t-test was carried out to compare among the clusters and corresponding results (P values) were recorded in **S7 Table**. The overall cellular enrichment pattern appeared highly consistent with the cluster pathway features (**Figs 4B & 7B**). Specifically, cluster 1 showed low level of fibroblasts and endothelial signatures, corresponding to the under-enriched Pathway module Blue, on the other hand, it had moderate enrichment in myeloid and macrophage that corresponds to Pathway module Red. Cluster 2 exhibited elevated level of myeloid + macrophages (Pathway module Red & Green) and moderate enrichment in fibroblasts + endothelial (Pathway module Blue). Cluster 3 only enriched in fibroblasts + endothelial which was reflected by Pathway module Blue in **Fig 4B**. Cluster 4, with strong expression in all 4 cell types, had elevated enrichment in Pathway modules Blue, Red and Green. In the similar manner, cluster 5, 6, 7 and 8 respectively displayed enrichment pattern of the 4 cell types that linked to 3 Pathway modules. In summary, Red and Green pathway groups are strongly associated with myeloid signature while Blue pathways correspond steadily with fibroblasts activity. The establishment of such cells-to-pathway affiliation also helped us defining the main biological functions of each pathway module (**Table 1**). In contrast to the previous studies which demonstrated differential expression levels of multiple immune cell lineage gene signatures, our cellular deconvolution demonstrated the prevalent myeloid cell signature among multiple SSc clusters, especially the co-variation between myeloid and macrophage gene signatures. The dysfunction of macrophage can lead to aberrant tissue repair and uncontrolled tissue modelling by communicating with epithelial cells, endothelial cells and fibroblasts by producing pro-fibrotic mediators and enhance the survival and activation of myofibroblasts. Hence, our analysis substantially underscored the contribution of myeloid cells to SSc pathogenesis.

## Discussion and conclusion

The current meta-analysis implemented novel computational approaches with the focus on deciphering new biological insights of SSc pathogenesis to compliment known mechanisms, and comprehensively characterized SSc disease subsets. Our analyses explored pathway driven meta-analysis by reducing the number of biological pathways from a comprehensive pathway database: Metabase^®^ using machine learning assisted feature selection. We picked Metabase^®^ over other pathways sources based on a balanced view between accurate coverage of immune related biological processes and avoidance of potentially redundant entries. The selected 80 pathways subjected to consensus clustering could reconstruct 8 SSc subtypes with comparative accuracy to that of all 693 pathways. Further analyses exploring topological network of 8 subtypes unveiled some key gene regulators—potentially as novel targets. *In-silico* cellular deconvolution was also conducted for each SSc subtype revealing the dominant immune and fibroblast components with the close association to each disease subtype. Our machine learning assisted clustering method allowed us identifying more pertinent SSc subtypes compared to previous studies, shedding light upon the development of immune-precision SSc treatment.

Previous SSc meta-analysis integrate multiple datasets with varying source effects by merging data together after normalization and baseline correction procedure [18]. We adopted an alternative approach by transforming individual gene expression into common pathway space using GSVA algorithm [22]; this step was followed by consensus clustering on the random forest classifier that elected an 80 pathway set. Our analysis identified 8 detailed SSc subtypes, revealing the heterogeneity of SSc and an objective data driven approach delineating the genomic differences that defined SSc pathogenesis. We were able to highlight 5 pathway modules that can differentiate the 8 clusters by their expression level combinations (**Fig 4B**). For instance, cluster 1 and 6 resembled the control cohort, showing active cellular metabolism pathways (Pathway module Black) similar to the normal control group. However, we noticed a subtle difference between the two subtypes that cluster 1 was actually mixed with elevated levels of immune response genes. Cluster 2 and 4 classified as Pathway module Blue, demonstrated moderate difference in terms of the extent of the enriched inflammation signature. Cluster 3, showing elevated levels in the Blue module only, formed a distinct cohort. Consistent with literature, Cluster 5 and 8, characterized as Pathway module Yellow exhibited a mixture of strong inflammatory, pro-fibrotic and cell-metabolism signatures. Lastly, cluster 7 appeared to be a separate “normal-like” cohort. Collectively, the 8 SSc cluster subtyping system was a more pertinent and finer stratification compared to the paradigmatic type classifications (inflammatory, normal-like and fibroproliferative) by previous literatures [21, 37, 38].

The 8 SSc subtypes we assessed in the study can be grouped to inflammatory, fibro-inflammatory, metabolic-fibrosis, metabolic, and normal-like phenotypes. For the inflammatory dominant clusters, such as cluster 2 and cluster 4, it is evident that IL17, IL22 pathways were highlighted, suggesting the hyperactive Th17 cells in such groups of patients. Current knowledge indicates complex roles of Th17 in fibrogenesis: One study demonstrated that IL-17 derived from Th17 cells promoted collagen production and fibroblast growth [39]. Another recent analysis showed that type 3 inflammation characterized by IL17 and IL22 by adaptive immune cells contribute to liver fibrosis [40]. Herein, cluster 4 is enriched for IL17/IL22 adaptive immune pathways together with IL-1 signaling pathway suggesting synergistic effects on the expression of pro-fibrotic and inflammatory mediators in dermal fibroblasts. Clusters 4, 5, and 8 of SSc patients in our meta-analysis have been attributed to the enrichment of biological pathways associated with the myeloid cells and fibrosis, such MCP1 and CD40 pathways. In the context of SSc, monocyte, macrophage, DCs including pDC either contribute indirectly to fibrogenesis in SSc by affecting inflammatory responses, or directly contribute to over-production of profibrotic factors and ECM proteins, such as TGFβ and collagen I [41]. In contrast to M1 macrophage, M2 macrophages are heavily involved in the process of tissue fibrosis. Abundant M2 macrophages infiltrated the lesional skin and lung tissues of SSc patients. pDCs is the major resource type 1 interferons production. Highly enriched IFN pathway in clusters 4, 5 and 8 highlights the importance of pDC contributing to fibrogenesis by over-production of type I IFNs. Metabolic reprogramming plays an instrumental role in regulating immune cell function and inflammatory responses, which are notable in the custers1 and 6 from our analyses. For the instance of SSc, glycolysis was significantly increased after fibroblast activation [42]. Actually, the significantly enriched oxidative phosphorylation metabolic pathways exert on macrophage polarizations to profibrogenic M2-like macrophage differentiation. Thus, although cluster 1 and cluster 5 SSc subtypes resemble to normal control group from some aspects, highly dysregulated metabolic profile of such subgroups of SSc patients suggests a profound metabolic reprograming contributing the pathological mechanisms of SSc. We also observed a group of SSc patients more resemble a normal-like pathway features. We speculate that cluster 6 patients with a normal-like transcript profile also tend to have milder disease with lower MRSS and lower skin score at the biopsy site as proposed by Assassi et al. [20]. It should be interesting to determine if there is a correlation of the decreasing composite fibroinflammatory score in this group of patients. Hence, the current meta-analysis will facilitate development of feasible biomarkers for intrinsic SSc subset classification with the goal to maximize the treatment benefit of certain therapies, and develop precision treatments of SSc.

Another distinct computational approach we adopted was to analyze the topology of PPI networks derived from each cluster using two algorithms: 1) random walk [32] and 2) neighborhood scoring [31]. By combining MRSS scores representative of SSc disease severity, our network analyses discovered hub genes that are significantly connected with start nodes while controlling for vertex promiscuity. A concentrated target list of 164 genes was generated with a few key genes such as CCL2, COL1A1, FGFR1, FN1, IGF1, IL6, IRAK2, MMP1 and TLR7 that were closely correlated with MRSS scores. It is noteworthy to highlight that these genes belong to a few important biological processes, such as cytokine signaling in the innate immune system, PI3K-Akt signaling pathway, extracellular matrix organization and TLR cascade. The results significantly strengthened the contribution of the innate immune system, in particular, myeloid cells, and fibroblasts potentially governing the pathological events in SSc. Of note, these hub genes demonstrated to a varying extent mRNA expression and may be identified in only one or at most two clusters of SSc patients; this was consistent with the contribution of such key genes to the SSc disease phenotypes we observed.

To expand the biological discoveries from our meta-analysis, *in silico* cellular deconvolution was applied to determine to what extent each immune or stromal cell subset is associated with the identified 8 disease subtypes. Our analyses showed that the SSc metabolic phenotype normally correlated with low enrichment of cellular components–except for cluster 8, which are enriched by all cellular components. The general moderate-to-low patterns of cell type enrichment shown in **Fig 7** suggest that the SSc subtypes with dominant metabolic pathways are associated with a “normal” like phenotype. In contrast, clusters 2, 3, 4, and 5, which are annotated as immune response dominant, exhibited substantially elevated levels of myeloid lineage enrichment. In particular, Cluster 4, with strong expression in all 4 cell types, has elevated enrichment in the Pathway module of pro-fibrotic and/or immune responses. In general, this analysis underscores the important involvement of myeloid cells, particularly macrophages, linked to fibroblast pathway genes. The establishment of such cells-to-pathway affiliation helped to define the main biological functions of each pathway module.

Our meta-analysis on SSc public studies, on the other hand, is not without limitations: Firstly, it has always been challenging to achieve enough sample size in meta-analysis of this type of rare human disease. In addition, there is a severe unbalanced distribution of lSSc and dSSc patient numbers in our analysis, which limited the power to delineate the molecular differences between the two categories. Thirdly, all the SSc public datasets available so far are based on microarray platforms that have limited resolution due to probe designs. Last and most importantly, the lack of important clinical information–e.g. disease duration or treatment history, in majority of the public datasets may hamper our effort in accurately defining the disease phenotypes. For example, some subtype of patients such as the normal-like group, may simply represent the early stage of the disease if we have disease duration data available. In view of these concerns, a more comprehensive future longitudinal study of SSc patients is needed to unveil critical biological mechanisms behind the etiology, disease progression, and prognostics of this dangerous autoimmune disorder.

In summary, our findings based on the current pathway driven meta-analysis indicate that there exists more complex disease structure in SSc than previously described. These results also confirmed the important role of myeloid cells in SSc pathogenesis. Nevertheless, the extent of contribution to disease phenotype through myeloid cell involvement still warrants further examination. Meanwhile, important hub genes identified through our module specific network analysis overlapped strongly with current clinical evidences; some of the genes are potential drug targets and may serve as the foundation for next generation of SSc treatment.

## Supporting information

S1 Checklist PRISMA 2009 checklist. (DOC)

S1 Fig 2- dimensional PCA plot showing pathway enrichment scores of all samples from 9 SSc studies. Samples are colored by study sources and clinical phenotypes are denoted in different shapes.
(TIF)

S1 Table Studies included in the current meta-analysis and their disease phenotype breakdown. Some samples are repeated at different time series points, before-after treatments or biopsy positions. Detailed inclusion or exclusion standards are specified in Method section. ^1^ Whitefield Lab from department of Genetics, Dartmouth Medical School, Hanover, NH, USA; ^2^ Assassi Group from University of Texas Health Science Center, Houston, TX, USA; ^3^ Bioinformatics Core facility, University of Manchester, Manchester, UK.
(DOCX)

S2 Table The SSc samples distribution (by counts) among 8 SSc clusters: Study names denoted by GEO numbers are in row header and Cluster number denoted by C1~C8 are in column header. *Percentages in the table represent total percentages.
(DOCX)

S3 Table Diffuse and limited SSc samples distribution (by counts) among 8 SSc clusters: Diffuse and limited SSc denoted by dSSc and lSSc are in row header and Cluster number denoted by C1~C8 are in column header. *Percentages in the table represent total percentages.
(DOCX)

S4 Table Pathway module enrichment levels among the 8 clusters and the control group. The average of pathway enrichment scores for each cluster & pathway-module combination from **Table 1** was calculated and shown.
(DOCX)

S5 Table Five pathway modules and their corresponding pathways in detail. Pathways modules identified from Fig 4B are shown in the left column and their corresponding pathways are listed in the right column.
(DOCX)

S6 Table List of 163 genes from network analysis overlapped with MRSS positively correlated genes. Pearson correlation coefficient and related p value are used to denote association between MRSS and gene expression, the algorithm sources (random walk or neighborhood scoring) and pathway module (1~5) are shown in column 1 and 2.
(XLSX)

S7 Table Cell type enrichment pairwise comparison among 8 SSC clusters and the control group. One-sided Welch T test was performed to compare cell type enrichment level among the 8 SSC clusters and the control group. P value matrix (corrected by Bonferroni procedure) was generated. The comparison was based on the alternative hypothesis that cluster in row label > cluster in column label
(XLSX)
