# Supplementary figures and images for: Transcriptional insights into pathogenesis of cutaneous systemic sclerosis using pathway driven meta-analysis assisted by machine learning methods
Source: PLoS One. 2020 Nov 30;15(11):e0242863. doi: 10.1371/journal.pone.0242863 (PMC7703909; doi:10.1371/journal.pone.0242863)

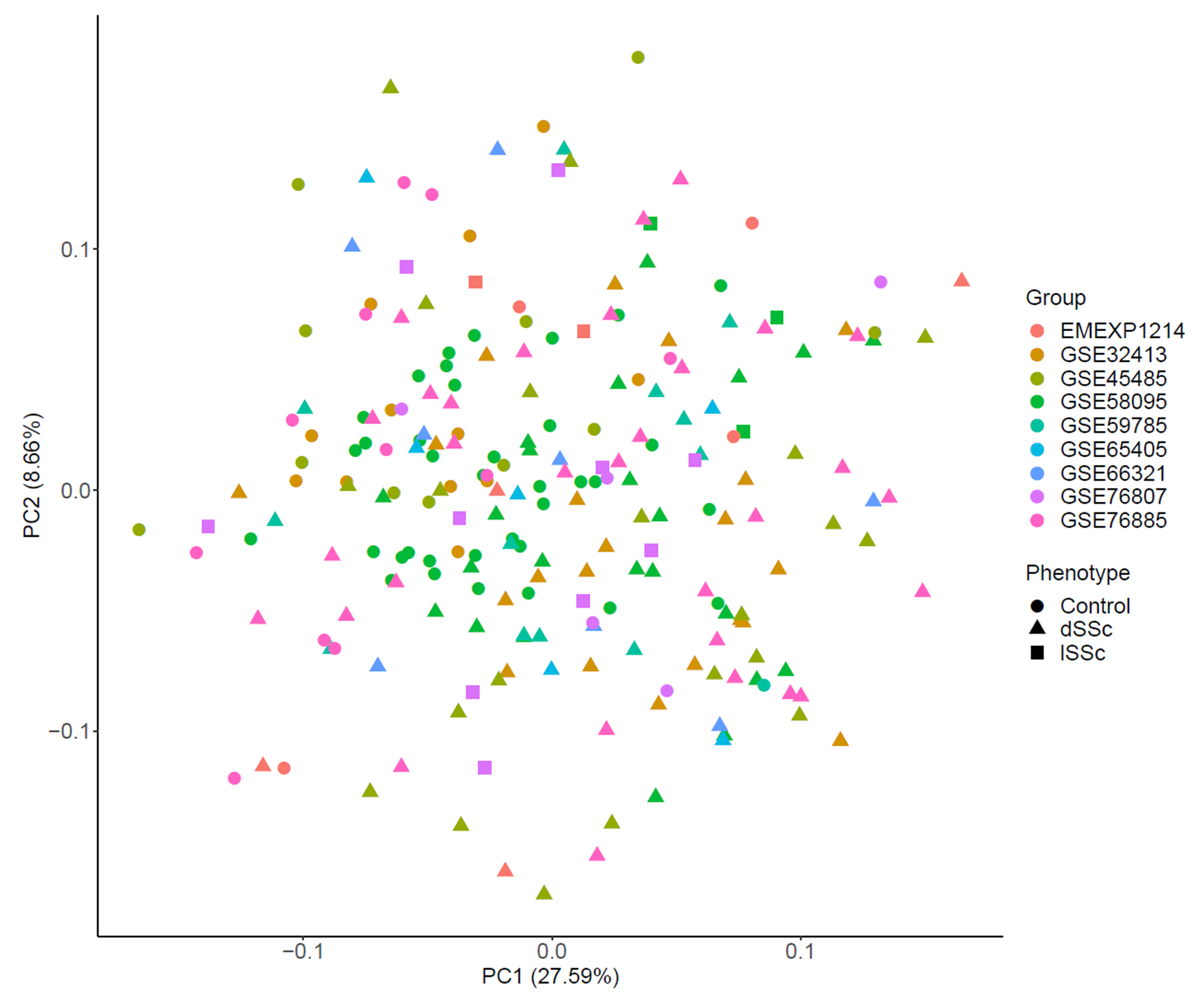

Supplement: S1 Fig — Samples are colored by study sources and clinical phenotypes are denoted in different shapes. (TIF) [file pone.0242863.s002.tif]
