# Supplementary material for: Transcriptional insights into pathogenesis of cutaneous systemic sclerosis using pathway driven meta-analysis assisted by machine learning methods
Source: PLoS One. 2020 Nov 30;15(11):e0242863. doi: 10.1371/journal.pone.0242863 (PMC7703909; doi:10.1371/journal.pone.0242863)
Supplement: S1 Table — Some samples are repeated at different time series points, before-after treatments or biopsy positions. Detailed inclusion or exclusion standards are specified in Method section. 1 Whitefield Lab from department of Genetics, Dartmouth Medical School, Hanover, NH, USA; 2 Assassi Group from University of Texas Health Science Center, Houston, TX, USA; 3 Bioinformatics Core facility, University of Manchester, Manchester, UK. (DOCX) [file pone.0242863.s003.docx]

| GEO Study | Total # of samples | | Patients class Breakdown | Source (Lab) | Comment |
| --- | --- | --- | --- | --- | --- |
| GSE32413 | 31 | 13 dSSc - ritum / 9 dSSc / 9 Ctrl | | Whitefield Lab^1^ | Rituximab treatment; Part of GSE76809 |
| GSE45485 | 32 | 11 Ctrl / 11 SScMH / 10 SSC | | Whitefield Lab | Part of GSE59787(Hinchcliff165), Mycophenolate mofetil treatment |
| GSE58095 | 102 | 36 Ctrl / 48 dSSc / 18 lSSc | | Assassi Group^2^ | Very detailed study, related to GSE47162 |
| GSE59785 | 24 | 1 Ctrl / 21 SSC-MH / 2 SSC | | Whitefield Lab | Part of GSE59787 (Hinchcliff 165), GSE76809 (Multitissue SSC) and GSE76886 (Hinchcliff 359) |
| GSE76885 | 66 | 11 Ctrl / 54 SSc / 1 morphea | | Whitefield Lab | Part of GSE76886, Hinchcliff 359 set |
| E-MEXP-1214 | 10 | 4 dSSc / 2 lSSc / 4 Control | | University of Manchester3 | Skin sample |
| GSE76807 | 15 | 10 lSSc / 5 Ctrl | | Whitefield Lab | Part of GSE76809, skin, lSSc and normal controls |
| GSE66321 | 8 | 8 SSc | | Whitefield Lab | An Abatacept vs placebo study |
| GSE65405 | 6 | 6 SSc | | Whitefield Lab | A Nilotinib treatment study |

**S1 Table**
