# Supplementary material for: Transcriptional insights into pathogenesis of cutaneous systemic sclerosis using pathway driven meta-analysis assisted by machine learning methods
Source: PLoS One. 2020 Nov 30;15(11):e0242863. doi: 10.1371/journal.pone.0242863 (PMC7703909; doi:10.1371/journal.pone.0242863)
Supplement: S2 Table — *Percentages in the table represent total percentages. (DOCX) [file pone.0242863.s004.docx]

**S2 Table:**

| Clusters* | C1 | | C2 | | C3 | | C4 | | C5 | | C6 | | C7 | | C8 | |  |
| --- | --- | --- | --- | --- | --- | --- | --- | --- | --- | --- | --- | --- | --- | --- | --- | --- | --- |
| EMEXP1214 | | 1 (0.7%) | | 0 (0.0%) | | 1 (0.7%) | | 1 (0.7%) | | 0 (0.0%) | | 2 (1.4%) | | 1 (0.7%) | | 0 (0.0%) | |
| GSE32413 | | 5 (3.5%) | | 3 (2.1%) | | 1 (0.7%) | | 5 (3.5%) | | 1 (0.7%) | | 1 (0.7%) | | 0 (0.0%) | | 5 (3.5%) | |
| GSE45485 | | 3 (2.1%) | | 3 (2.1%) | | 2 (1.4%) | | 6 (4.3%) | | 1 (0.7%) | | 3 (2.1%) | | 2 (1.4%) | | 1 (0.7%) | |
| GSE58095 | | 9 (6.4%) | | 4 (2.8%) | | 3 (2.1%) | | 2 (1.4%) | | 8 (5.7%) | | 0 (0.0%) | | 0 (0.0%) | | 0 (0.0%) | |
| GSE59785 | | 2 (1.4%) | | 1 (0.7%) | | 3 (2.1%) | | 0 (0.0%) | | 2 (1.4%) | | 2 (1.4%) | | 0 (0.0%) | | 2 (1.4%) | |
| GSE65405 | | 2 (1.4%) | | 1 (0.7%) | | 0 (0.0%) | | 0 (0.0%) | | 0 (0.0%) | | 1 (0.7%) | | 0 (0.0%) | | 2 (1.4%) | |
| GSE66321 | | 1 (0.7%) | | 1 (0.7%) | | 1 (0.7%) | | 1 (0.7%) | | 0 (0.0%) | | 2 (1.4%) | | 1 (0.7%) | | 1 (0.7%) | |
| GSE76807 | | 1 (0.7%) | | 0 (0.0%) | | 2 (1.4%) | | 0 (0.0%) | | 0 (0.0%) | | 2 (1.4%) | | 2 (1.4%) | | 3 (2.1%) | |
| GSE76885 | | 4 (2.8%) | | 6 (4.3%) | | 4 (2.8%) | | 4 (2.8%) | | 3 (2.1%) | | 4 (2.8%) | | 2 (1.4%) | | 4 (2.8%) | |
| Total (N = 141) | | 28 (19.9%) | | 19 (13.5%) | | 17 (12.1%) | | 19 (13.5%) | | 15 (10.6%) | | 17 (12.1%) | | 8 (5.7%) | | 18 (12.8%) | |
