# Supplementary material for: Transcriptional insights into pathogenesis of cutaneous systemic sclerosis using pathway driven meta-analysis assisted by machine learning methods
Source: PLoS One. 2020 Nov 30;15(11):e0242863. doi: 10.1371/journal.pone.0242863 (PMC7703909; doi:10.1371/journal.pone.0242863)
Supplement: S3 Table — *Percentages in the table represent total percentages. (DOCX) [file pone.0242863.s005.docx]

**S3 Table:**

| **Clusters*** | **dSSc** | **lSSc** | **Grand Total** |
| --- | --- | --- | --- |
| **C1** | 27 (19.1%) | 1 (0.7%) | 28 (19.9%) |
| **C2** | 19 (13.5%) | 0 (0.0%) | 19 (13.5%) |
| **C3** | 14 (9.9%) | 3 (2.1%) | 17 (12.1%) |
| **C4** | 19 (13.5%) | 0 (0.0%) | 19 (13.5%) |
| **C5** | 12 (8.5%) | 3 (2.1%) | 15 (10.6%) |
| **C6** | 14 (9.9%) | 3 (2.1%) | 17 (12.1%) |
| **C7** | 6 (4.3%) | 2 (1.4%) | 8 (5.7%) |
| **C8** | 15 (10.6%) | 3 (2.1%) | 18 (12.8%) |
| **Total** | 126 (89.4%) | 15 (10.6%) | 141 (100.0%) |
