# Supplementary material for: Transcriptional insights into pathogenesis of cutaneous systemic sclerosis using pathway driven meta-analysis assisted by machine learning methods
Source: PLoS One. 2020 Nov 30;15(11):e0242863. doi: 10.1371/journal.pone.0242863 (PMC7703909; doi:10.1371/journal.pone.0242863)
Supplement: S4 Table — The average of pathway enrichment scores for each cluster & pathway-module combination from Table 1 was calculated and shown. (DOCX) [file pone.0242863.s006.docx]

**S4 Table:**

| Clusters \ Modules | Black | Yellow | Blue | Red | Green |
| --- | --- | --- | --- | --- | --- |
| Cluster 1 | 0.323 | 0.219 | -0.346 | 0.003 | -0.113 |
| Cluster 2 | -0.301 | 0.102 | 0.009 | 0.357 | 0.294 |
| Cluster 3 | -0.376 | 0.008 | 0.237 | -0.108 | -0.002 |
| Cluster 4 | -0.371 | -0.277 | 0.335 | 0.381 | 0.383 |
| Cluster 5 | 0.299 | 0.243 | 0.317 | 0.288 | 0.267 |
| Cluster 6 | 0.258 | 0.287 | -0.306 | -0.378 | -0.333 |
| Cluster 7 | -0.289 | 0.254 | -0.299 | 0.103 | -0.155 |
| Cluster 8 | 0.121 | -0.193 | 0.188 | 0.189 | 0.212 |
| Control | 0.115 | 0.008 | -0.009 | -0.192 | -0.185 |
