# Supplementary material for: Transcriptional insights into pathogenesis of cutaneous systemic sclerosis using pathway driven meta-analysis assisted by machine learning methods
Source: PLoS One. 2020 Nov 30;15(11):e0242863. doi: 10.1371/journal.pone.0242863 (PMC7703909; doi:10.1371/journal.pone.0242863)
Supplement: S5 Table — Pathways modules identified from Fig 4B are shown in the left column and their corresponding pathways are listed in the right column. (DOCX) [file pone.0242863.s007.docx]

**S5 Table:**

| **Pathway Modules** | **Pathways** |
| --- | --- |
| Black | Tricarbonic acid cycle  Saturated fatty acids synthesis to hexadecanoic acid  Mitochondrial beta oxidation of long chain saturated fatty acids  Regulation of lipid metabolism PPAR regulation of lipid metabolism  Mitochondrial unsaturated fatty acid beta oxidation  Pyruvate metabolism  Cholesterol Biosynthesis  Mitochondrial ketone bodies biosynthesis and metabolism  Butanoate metabolism  Propionate metabolism p 2  Peroxysomal straight chain fatty acid beta oxidation  Unsaturated fatty acid biosynthesis  n 3 Polyunsaturated fatty acid biosynthesis  n 6 Polyunsaturated fatty acid biosynthesis |
| Yellow | CFTR translational fidelity class I mutations  Translation L-selenoamino acids incorporation in proteins during translation  Cortisol biosynthesis from Cholesterol  Cortisone biosynthesis and metabolism  Regulation of lipid metabolism FXR dependent negative feedback regulation of bile acids concentration  Development Insulin IGF 1 and TNF alpha in brown adipocyte differentiation  Regulation of metabolism Bile acids regulation of glucose and lipid metabolism via FXR |
| Blue | Development TGF beta dependent induction of EMT via MAPK  Chemotaxis SDF 1 CXCR4 induced chemotaxis of immune cells  CCR7 signaling pathways in dendritic cells in allergic contact dermatitis  Immune response MIF induced cell adhesion migration and angiogenesis  Immune response CCL2 signaling  G protein signaling G Protein alpha 12 signaling pathway  Development EPO induced MAPK pathway  Development SDF 1 signaling in hematopoietic stem cell homing  Immune response IFN gamma signaling pathway  Chemotaxis CCL19 and CCL21 mediated chemotaxis  Immune response Histamine H1 receptor signaling in immune response  Oxidative stress Role of IL 8 signaling pathway in respiratory burst  Immune response T regulatory cell mediated modulation of effector T cell and NK cell functions  Substance P mediated inflammation and pain in Sickle cell disease  Immune response PIP3 signaling in B lymphocytes  Immune response Mast cell proliferation differentiation and survival  Role of CNTF and LIF in regulation of oligodendrocyte development in multiple sclerosis  Apoptosis and survival BAD phosphorylation  Development HGF signaling pathway  wtCFTR and deltaF508 CFTR traffic Generic schema normal and CF  Development Regulation of cytoskeleton proteins in oligodendrocyte differentiation and myelination  Cytoskeleton remodeling Fibronectin binding integrin’s in cell motility  Glucocorticoid induced elevation of intraocular pressure as glaucoma risk factor |
| Red | Rheumatoid arthritis general schema  Role of IL 17 producing T cells in allergic contact dermatitis  Immune response T cell co signaling receptors schema  NK cells in allergic contact dermatitis  Renal tubule-interstitial injury in Lupus Nephritis  Role of B cells in SLE  Maturation and migration of dendritic cells in skin sensitization  Role of keratinocytes and Langerhans cells in skin sensitization  LRRK2 and immune function in Parkinson s disease  Immune response Generation of memory CD4 T cells  Immune response Th1 and Th2 cell differentiation  Immune response IL 22 signaling pathway  Immune response Differentiation of natural regulatory T cells  Immune response NF AT signaling and leukocyte interactions  Immune response NF AT in immune response  T follicular helper cell dysfunction in SLE  Immune response |
| Green | Complement pathway disruption in thrombotic microangiopathy  iNKT cell keratinocyte interactions in allergic contact dermatitis  Role of Bregs in attenuation of T and NK cells mediated anti-tumor immune responses  Cell adhesion Integrin inside out signaling in T cells  Immune response Immunological synapse formation  PDE4 regulation of cyto chemokine expression in arthritis  Immune response CD40 signaling  Immune response Role of PKR in stress induced antiviral cell response  Immune response Bacterial infections in normal airways  B regulatory cells and tumor cells intercellular interaction  Bacterial infections in CF airways  SLE genetic marker specific pathways in B cells  Cell adhesion Integrin inside out signaling in neutrophils  Inhibition of neutrophil migration by pro-resolving lipid mediators in COPD  Immune response IL 12 induced IFN gamma production  Immune response HSP60 and HSP70 TLR signaling pathway  Immune response IL 5 signaling via PI3K MAPK and NF kB  Immune response IL 18 signaling |
